# Loss of SPRR3 in *ApoE*^-/-^ mice leads to atheroma vulnerability through Akt dependent and independent effects in VSMCs

**DOI:** 10.1371/journal.pone.0184620

**Published:** 2017-09-08

**Authors:** Caressa D. Lietman, Amanda K. Segedy, Bin Li, Sergio Fazio, James B. Atkinson, MacRae F. Linton, Pampee P. Young

**Affiliations:** 1 Department of Pathology Microbiology and Immunology; Vanderbilt University Medical Center; Nashville, TN, United States of America; 2 Center of Preventive Cardiology; Knight Cardiovascular Institute; Oregon Health & Science University; Portland, OR, United States of America; 3 Veterans Affairs Medical Center, Nashville, TN, United States of America; 4 Department of Pharmacology, Vanderbilt University Medical Center; Nashville, TN, United States of America; 5 Department of Medicine; Vanderbilt University Medical Center; Nashville, TN, United States of America; Qatar University College of Health Sciences, QATAR

## Abstract

Vascular smooth muscle cells (VSMCs) represent important modulators of plaque stability in advanced lesions. We previously reported that loss of small proline-rich repeat protein 3 (*Sprr3*), leads to VSMC apoptosis in a PI3K/Akt-dependent manner and accelerates lesion progression. Here, we investigated the role of *Sprr3* in modulating plaque stability in hyperlipidemic *ApoE*^-/-^ mice. We show that loss of *Sprr3* increased necrotic core size and reduced cap collagen content of atheromas in brachiocephalic arteries with evidence of plaque rupture and development of intraluminal thrombi. Moreover, *Sprr3*^-/-^*ApoE*^-/-^ mice developed advanced coronary artery lesions accompanied by intraplaque hemorrhage and left ventricle microinfarcts. SPRR3 is known to reduce VSMC survival in lesions by promoting their apoptosis. In addition, we demonstrated that *Sprr3^-/-^* VSMCs displayed reduced expression of procollagen in a PI3K/Akt dependent manner. SPRR3 loss also increased MMP gelatinase activity in lesions, and increased MMP2 expression, migration and contraction of VSMCs independently of PI3K/Akt. Consequently, *Sprr3* represents the first described VSMC modulator of each of the critical features of cap stability, including VSMC numbers, collagen type I synthesis, and protease activity through Akt dependent and independent pathways.

## Introduction

Plaque rupture is the most important mechanism underlying the sudden plaque progression that is responsible for acute coronary syndrome [1–4]. Contrary to common thought, data collected from autopsy studies and carotid endarterectomies indicates that plaque rupture does not occur as a consequence of narrowing of the lumen but is induced by a structural defect or gap in the fibrous cap, followed by intraluminal thrombosis [1, 5–7]. Studies of the mechanisms behind plaque rupture are limited, because the most commonly used mouse model of hyperlipidemia, atherosclerosis initiation, and atheroma development, the Apolipoprotein E (*ApoE*) deficient mice, rarely exhibit plaque destabilization and rupture [1–4]. Hence, good murine models of plaque rupture will not only help us better understand the etiology of plaque vulnerability, but may identify molecular targets for treatment.

Plaques vulnerable to rupture are characterized by a large lipid-rich core [8] and thin fibrous cap comprised mainly of collagen, which is usually secondary to reduced extracellular matrix production in vascular VSMC-poor caps [9, 10]. The fibrous cap consists mostly of acellular matrix and VSMCs, which are the primary cells that synthesize collagen, the major matrix constituent, as well as other matrix proteins. Fibrous cap thinning is hypothesized to result from matrix remodeling action driven by matrix metallopeptidases (MMPs) released by both VSMCs and macrophages and by decreased matrix synthesis for cap maintenance [10]. In fact, a growing number of studies point to a critical role for VSMC apoptosis/survival within the fibrous cap in modulating plaque vulnerability [11, 12]. Global deletion of *Akt1* in mice has been shown to reduce VSMC migration, proliferation, and survival and has been linked to features of plaque progression and instability, including enlarged necrotic core, reduced fibrous plaque, and even spontaneous infarcts, a feature rarely observed in murine models of atherosclerosis [11]. In contrast, VSMC specific deletion of *Akt1* in mice demonstrates a milder phenotype without evidence of rupture or myocardial infarct, suggesting that the effects of *Akt1* deletion are not solely mediated by VSMC expression [13]. To our knowledge no VSMC-specific factors implicated in regulating cap composition and plaque rupture have been identified [14]. Identification of proteins that act upstream of PI3K/Akt to regulate its activity in atheroma VSMCs would clearly represent an important advance.

SPRR3 is a member of the family of Small Proline-Rich Repeat proteins that was originally identified as heavily enriched in the esophagus, and possesses glutamine- and lysine-rich head and tail domains and a proline-rich core [15, 16]. Based on these sequence characteristics, SPRRs are presumed to covalently link structural proteins and/or each other by ε-(γ-glutamyl) lysine isopeptide bonds, although there are no *in vivo* data to support this and the precise cellular role(s) of the SPRR proteins has not been experimentally investigated [17, 18]. We serendipitously discovered that SPRR3 is highly expressed in human and mouse atheroma-associated VSMCs but not in healthy vasculature [19, 20]. SPRR3 expression was not detected in lung, liver, brain, or skeletal muscle [21]. Robust SPRR3 expression by immunohistochemistry has been detected in atheroma VSMCs but not VSMCs in unaffected portions of the vessels [15]. Moreover, we demonstrated that *Sprr3* loss results in plaque progression, at least in part, by enhancing VSMC apoptosis in lesions in a PI3K/Akt dependent manner [15, 20–22]. Interestingly, *Sprr3* has been noted to be upregulated in breast, brain, and colon cancer, where its increased expression has been correlated with enhanced epithelial cell proliferation, Akt and MDM2 activation, and downregulation of p53 via unknown mechanisms [23–25]. In this study, we demonstrate that *Sprr3* loss in *ApoE-*deficient mice leads to features of unstable lesions in the aortic root, brachiocephalic, and coronary arteries, with evidence of rupture and subsequent thrombus formation, along with spontaneous microinfarcts in the left ventricle of the heart. In addition to enhancing VSMC apoptosis, we demonstrate that *Sprr3* regulates other key VSMC functions that dictate cap strength, such as matrix synthesis and remodeling. To our knowledge, no previous study has identified a potential regulator of VSMC collagen synthesis, and SPRR3 represents the first protein identified to regulate the key features of atheroma cap stability including VSMC number, collagen synthesis and matrix degradation.

## Results

### *Sprr3*^-/-^*ApoE*^-/-^ mice exhibit increased vulnerable plaques with evidence of spontaneous, intraluminal thrombus formation

**Fig 1:**
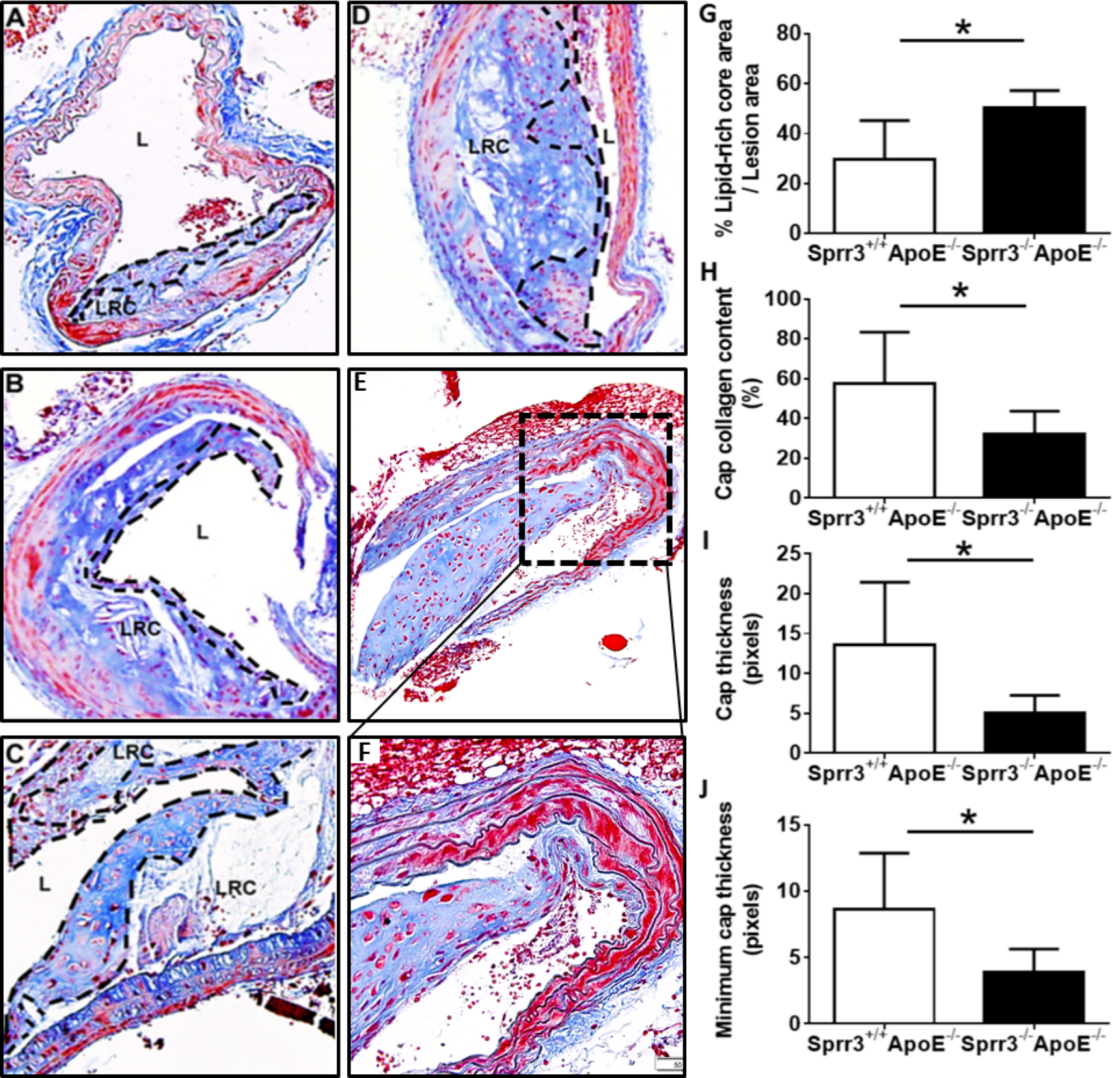
Advanced *Sprr3*^-/-^ brachiocephalic atheromas have reduced cap collagen content and thickness, larger lipid-rich cores and evidence of organizing thrombus. Brachiocephalic artery tissue sections collected from *Sprr3*^+/+^*ApoE*^-/-^ (A) or *Sprr3*^-/-^*ApoE*^-/-^ (B-F) mice fed high fat diet for 6 months (A-D; Masson’s trichrome; E-F; H&E). Features of plaque vulnerability were increased in the *Sprr3*^-/-^*ApoE*^-/-^ lesions, including evidence of an organized thrombus, which remained connected to endothelium, indicating that this was not an embolic thrombus (E-F). Other features such as larger lipid rich cores (LRC), reduced cap collagen content, and reduced cap thickness were also increased with SPRR3 loss and these data were quantified and shown graphically (G-I). L = lumen. Dashed line indicates lesion cap. Original magnification, x20 (A-F). * p < 0.05.

**Fig 2:**
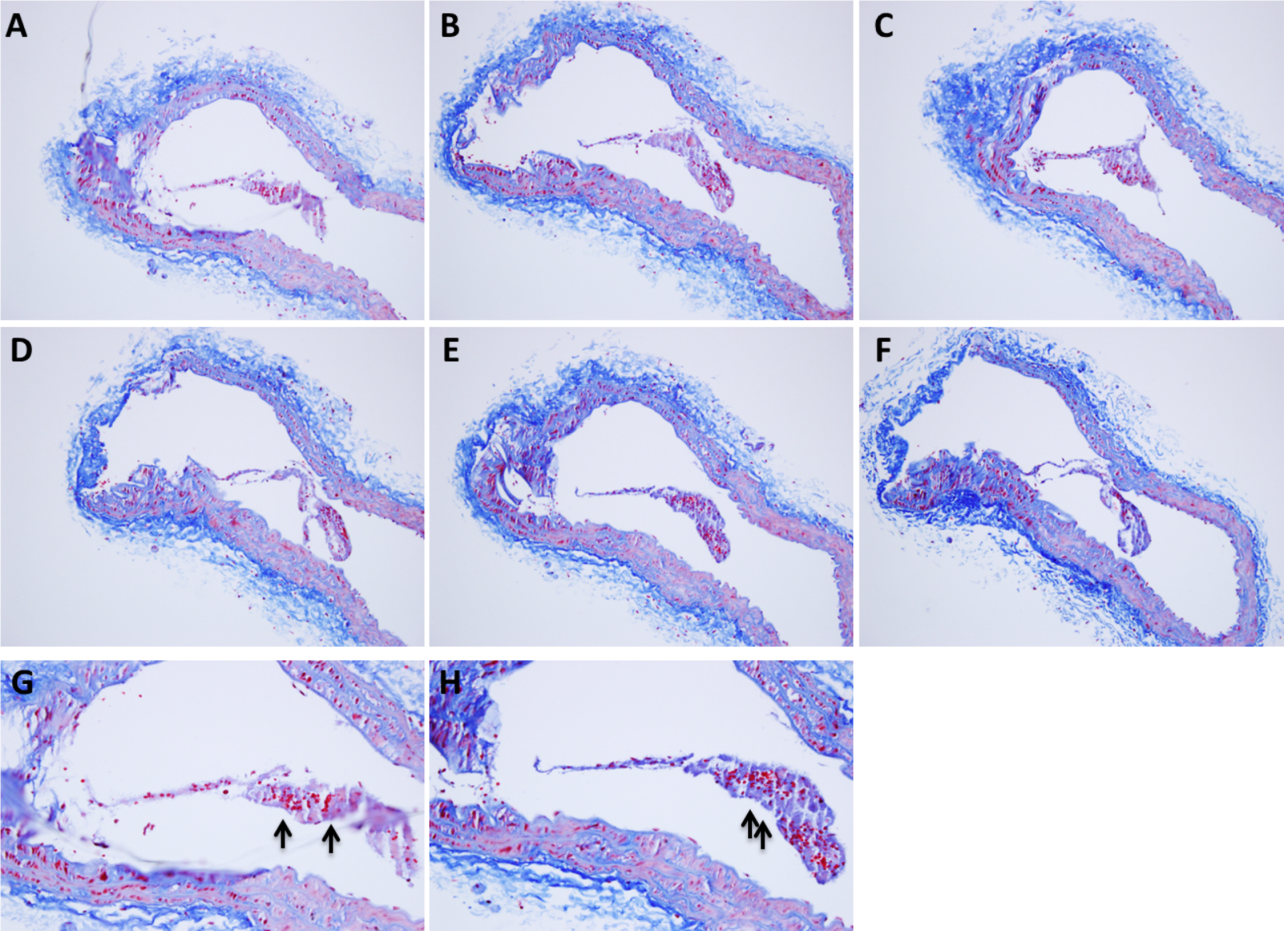
Intraluminal plaque-associated thrombi in *Sprr3*^-/-^*ApoE*^-/-^ mice. Examples (A-F, 10x) of intraluminal mural thrombi in DKO mice generated from areas of ruptured plaques. Higher magnification (G-H) images from two of these animals demonstrate characteristic intra-thrombi “lines of Zahn” that indicate formation prior to sacrifice (arrows, 40x).

Our previous work demonstrated reduced VSMC content and increased TUNEL-positive VSMCs in aortic root lesions of DKO mice when compared with those found in *ApoE*-null mice [20]. Moreover, to determine the role of SPRR3 loss in atheroma vulnerability, brachiocephalic artery sections were collected from *ApoE*-null (*Sprr3*^+/+^*ApoE*^-/-^, n = 12) and DKO (*Sprr3*^-/-^*ApoE*^-/-^, n = 12) mice fed high-fat diet for 6 months. *Sprr3*^-/-^ mice were primarily evaluated on the *ApoE*^-/-^ background to induce hyperlipidemia and atherosclerosis progression. It was previously shown that *Sprr3* ablation alone is not sufficient to generate significant atherosclerosis due to low blood lipid levels [26]. In prior studies, we demonstrated atheroma-restricted expression of SPRR3 in atheromas in various human arteries and in murine aortic root lesions, but not in lesion-free areas of blood vessels. As expected, *Sprr3* was also demonstrated in association with lesions in brachiocephalic and coronary lesions in the *ApoE*-null mice by immunofluorescence (S1 Fig). In the current study, DKO mice exhibited significantly larger lipid-rich cores compared to *ApoE*-null (as percent of lesion area: 50.4 ± 2.8% in DKO vs. 36.6 ± 5.4% in *ApoE*-null mice; Fig 1G, p<0.05). Percent cap collagen content was reduced in DKO mice when compared with *ApoE*-null controls (32.3 ±4.1% in DKO vs. 57.7 ± 12.8% in *ApoE*-null; Fig 1H, p<0.05). Cap area (thickness), when controlled for lesion area, was also reduced in DKO mice (0.08 ± 0.03 in DKO vs. 0.24 ± 0.02 in *ApoE*-null; Fig 1I, p<0.05). We also evaluated features of plaque instability in aortic roots (Figure A in S2 Fig n = 10). The DKO aortic root plaques had smaller fibrous cap area per plaque area with reduced collagen composition within the plaque compared to *ApoE*-null (Figures B-C in S2 Fig, n = 10). Evidence of spontaneous intraluminal thrombus is extremely rare in the mouse brachiocephalic artery [27]. We performed detailed histologic evaluation of brachiocephalic arteries from n = 24 mice in search of histologic evidence of plaque rupture. An example of an organized, intraluminal thrombus within the brachiocephalic artery found exclusively in the DKO mice is shown (Fig 1E and 1F). This intraluminal thrombus, which remained connected to the endothelium, showed extensive reorganization with evidence of fibrous connective tissue and blood vessels. Additional examples of newly-generated mural thrombi demonstrating connection to the endothelium and adjacent to ruptured lesion in brachiocephalic arteries of DKO mice were also identified in six DKO mice (Fig 2A–2F). The presence of alternating layers or laminations of red blood cells mixed with fibrin evident within the thrombi, the so-called “lines of Zahn”, suggest that these thrombi were created in the presence of vascular flow and were not likely generated post-mortem [28]. The demonstration of such intraluminal thrombi associated with plaques in mice is highly unusual.

A role for macrophages, particularly within the cap, has long been proposed as a contributing factor to plaque vulnerability [1]. Immunohistochemistry for F480, a macrophage marker, was performed on brachiocephalic artery sections from 6-month-old, high-fat fed *ApoE*-null (n = 10) and DKO (n = 10) mice (Figures A-B in S3 Fig). When evaluated for F480 positive cap area, no significant difference was observed between groups (1.7 ± 0.4% *Sprr3*^+/+^*ApoE*^-/-^, 1.5 ± 0.4% *Sprr3*^-/-^*ApoE*^-/-^, p = 0.7; S3C Fig). The absence of differences in macrophage density as a potential explanation for features of plaque instability is consistent with our findings that SPRR3-modulation of atheroma progression was independent of inflammatory cells [20]. Plasma levels of IL-1β (18±4 vs. 16.5±3 pg/ml, n = 6) and IL-6 (230±48 vs. 210±53 pg/ml, n = 6) were assessed by ELISA and not found to be statistically different between *ApoE*-null and DKO, respectively. These data indicate that characteristics of vulnerable plaques are increased in both advanced brachiocephalic atheromas and aortic roots of DKO mice when compared with *ApoE*-null controls without evidence of increased inflammation. Moreover, histologic evidence of plaque-associated thrombi was evident in DKO animals but not in *ApoE-*null animals.

Intraluminal thrombi in DKO mice may be potentially mediated by altered platelet activation or coagulation (i.e. via upregulation of tissue factor). To assess these possibilities, we evaluated platelet count (1.2x10^6^/ul±200 vs. 1.0x10^6^/ul±380; n = 6) and bleeding time (49±17 vs. 52±23 seconds, n = 5) in DKO vs. *ApoE*-null mice, respectively, and found no statistical difference in either parameter. To rule out altered expression of tissue factor as a mechanism of increased predisposition to thrombosis, we evaluated the expression of tissue factor on endothelial cells and VSMCs from *ApoE*-null vs. DKO mice (S4 Fig). As expected, the cell surface expression of tissue factor was higher on VSMCs than endothelial cells, which exhibited little to no detection of tissue factor. The cell surface expression of tissue factor on DKO VSMCs was slightly less than those from *ApoE*-null (S4 Fig, cells from n = 3 isolations).

### *Sprr3*^-/-^*ApoE*^-/-^ mice develop significantly more advanced coronary artery atherosclerotic lesions that exhibit features of plaque instability

**Fig 3:**
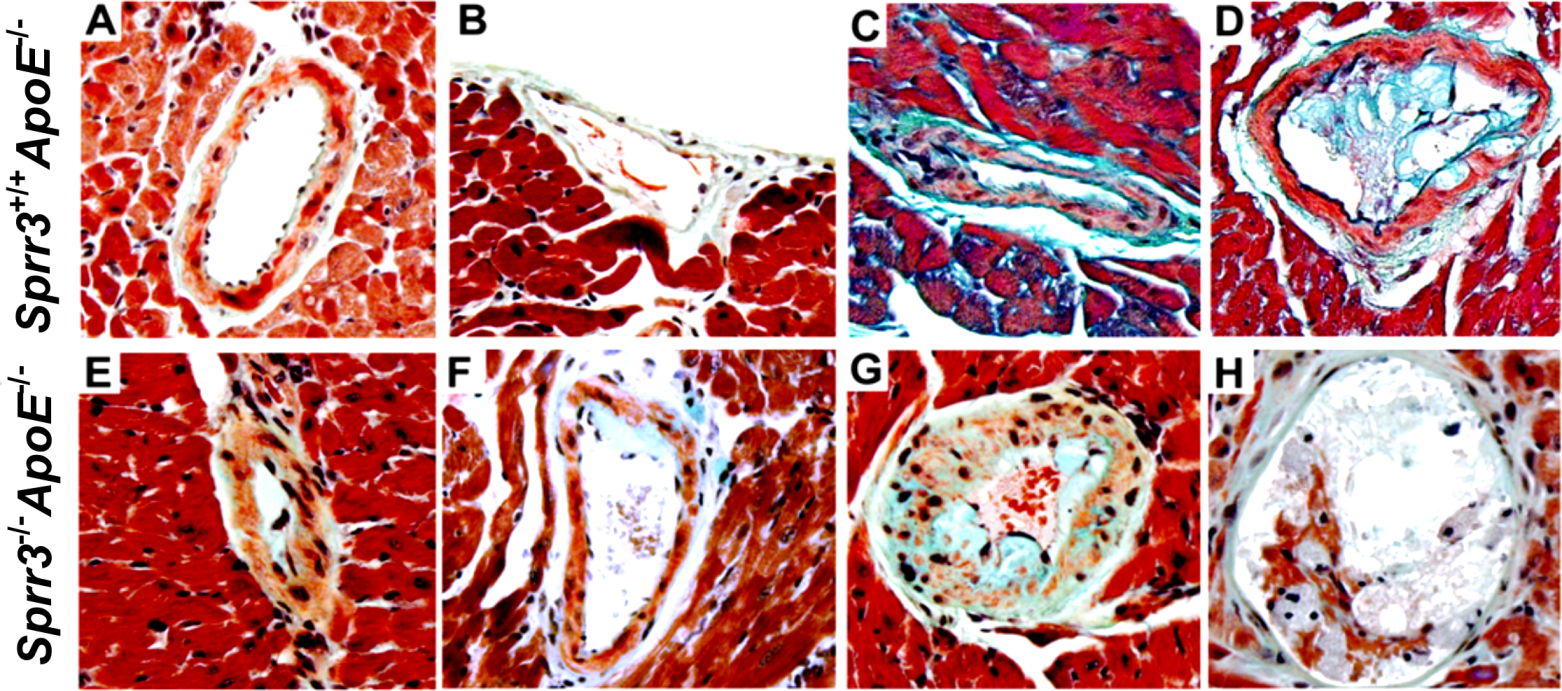
*Sprr3*^-/-^*ApoE*^-/-^ mice develop more advanced coronary artery atherosclerosis with evidence of complications. Left ventricular tissue sections collected from cardiac apices of *Sprr3*^+/+^*ApoE*^-/-^ or *Sprr3*^-/-^*ApoE*^-/-^ mice (n = 15) fed high fat diet for 6 months were stained with MOVAT pentachrome. Representative images are shown for arteries from both *ApoE* null (top row) and DKO (bottom row) mice containing no lesion (A), fatty streaks (B, E), intermediate lesion (C, F), advanced atheroma (D, G). Only DKO mice had coronary artery lesions with evidence of intraplaque hemorrhage (H). Original magnification, 40x.

Coronary lesions leading to plaque rupture represent the leading cause of myocardial infarctions (MIs) and death, but coronary lesions have rarely been reported or described in murine atherosclerosis models [27]. To determine the effect of *Sprr3* loss on the development of coronary artery atherosclerosis, cardiac apex sections from *ApoE*-null (n = 15) and DKO (n = 15) mice fed high fat diet for 6 months were stained with MOVAT and assessed for coronary artery atherosclerosis. To allow for sufficient space to distinguish features of atheroma progression, only arteries larger than 25 micrometers were included in the quantification. Atheroma burden in coronary arteries was classified according to severity as (1) no lesion, (2) early atheroma (including fatty streak and intermediate lesions), or (3) advanced atheroma. Both cohorts of animals had vessels containing lesions ranging from normal to advanced atheroma (Table 1). DKO mice had almost 6-fold more advanced atheromas in their coronary arteries compared to *ApoE*-null (DKO = 17.8 ± 7 and *ApoE*-null = 3.3 ± 5.3; p = 0.013), although no significant difference was noted in the number of lesion-free coronary arteries, fatty streaks or early atheromas (Table 1). Fig 3 provides representative photomicrographs of such lesions in *ApoE*-null and DKO mice (A-F). Of note, only DKO mice had coronary lesions with intraplaque hemorrhage (Fig 3G). Intrusion of erythrocytes into the plaque provided indirect evidence of plaque rupture [27]. These findings also reinforced our previous conclusion that *Sprr3* loss has direct effects on atheroma progression and plaque vulnerability without affecting plaque initiation.

**Table 1:** Loss of SPRR3 leads to increased advanced coronary artery atheromas.

	*ApoE*^-/-^*Sprr3*^+/+^	*ApoE*^-/-^*Sprr3*^-/-^
No lesion	80.5%±18.8	72.4%±11.5
Early atheroma	14.5%±9.7	15.2%±9
Advanced atheroma	3.3%±5.2	17.8%±7*

*Denotes statistical significance with p≤0.05

### *Sprr3*^-/-^*ApoE*^-/-^ mice develop evidence of left ventricular (LV) microinfarcts

**Fig 4:**
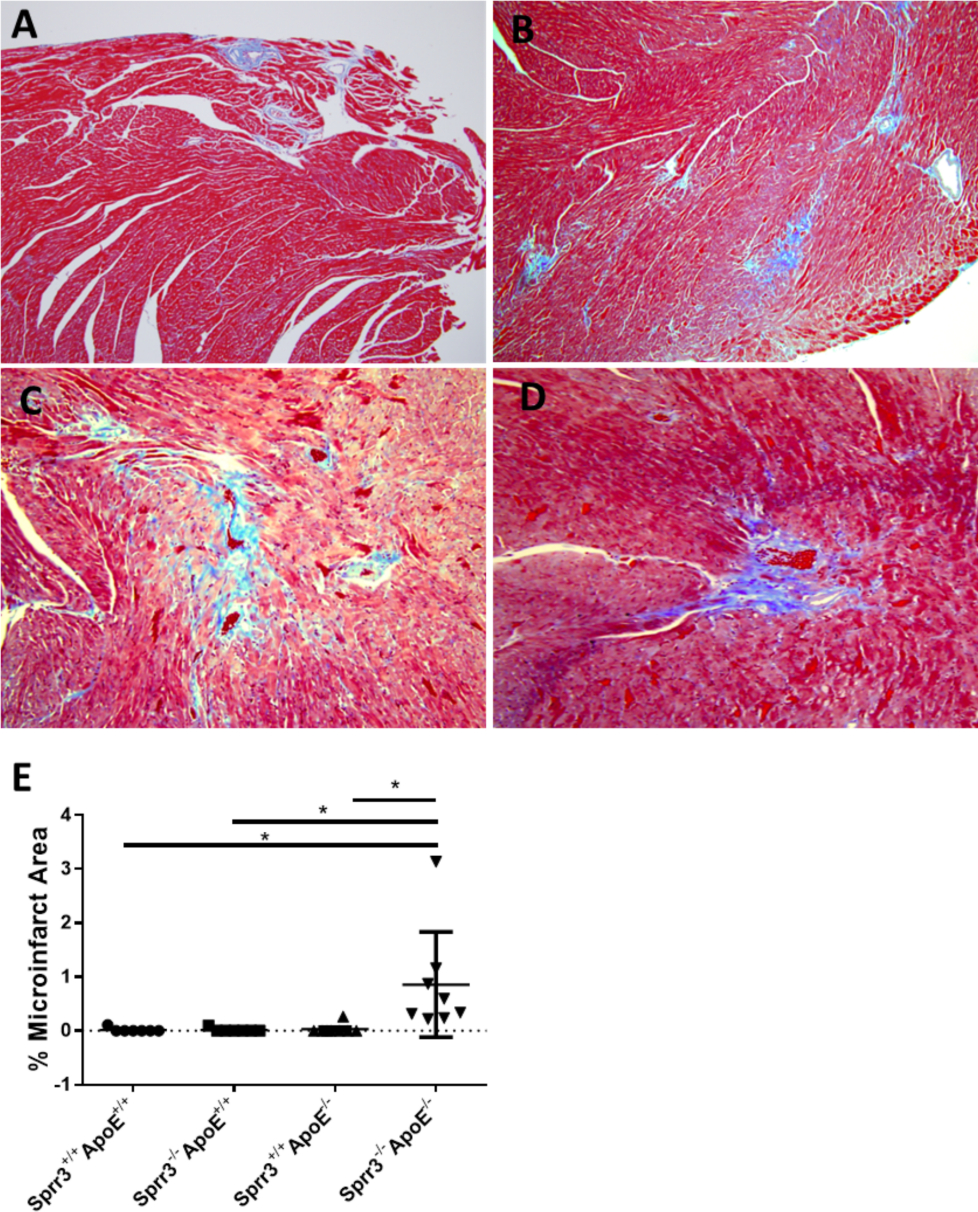
Loss of *Sprr3* in *ApoE*^-/-^ mice leads to spontaneous cardiac left ventricular infarctions. (A-D) Representative images of left ventricular histologic sections collected from *Sprr3*^-/-^*ApoE*^-/-^ (DKO) mice fed high fat diet for 6 months stained with Masson’s trichrome blue are shown. Small infarcts were observed in *Sprr3*^-/-^*ApoE*^-/-^ DKO mice. (E) Quantification of percent infarcted area in *Sprr3*^+/+^*ApoE*^+/+^, *Sprr3*^-/-^*ApoE*^+/+^, *Sprr3*^+/+^*ApoE*^-/-^ versus *Sprr3*^-/-^*ApoE*^-/-^ (n = 8 per group) mice. Original magnification, 10x. *p<0.05.

Since our evaluation of DKO brachiocephalic and coronary lesions exhibited features of unstable plaques as well as evidence of a significant increase in advanced coronary lesions, we sought to evaluate if loss of *Sprr3* in the *ApoE*-null atherogenic background caused myocardial infarctions and diminished cardiac function. Histological analysis of left ventricular (LV) tissue sections from DKO mice fed high fat diet for 6 months showed regions of scarring typical of healed ischemic microinfarcts, whereas no evidence of microinfarcts were found in wildtype, *ApoE*-null or *Sprr3*^-/-^*ApoE*^+/+^ control tissue sections (Fig 4A–4D, data graphed in 4E). To determine if LV function was compromised in DKO mice over *ApoE*-null, echocardiography was performed at 2 months of age (prior to start of HFD) and after 6 months of HFD (Table 2). Average left ventricle internal dimension diastolic (LVIDD) and LV internal dimension systolic (LVIDS) reflect remodeling in the infarcted ventricle were not significantly different. Similarly, there was no significant difference resulting from loss of *Sprr3* on cardiac function as measured by ejection fraction (EF). Given the size of the infarcts, the absence of any effects on cardiac function was not unexpected.

**Table 2:** Loss of *Sprr3* did not significantly impact LV dimensions or function after 6 months of HFD.

Parameters	*ApoE* ^-/-^*Sprr3*^+/+^		*ApoE* ^-/-^*Sprr3*^-/-^		P value
	Base	6M HFD	Base	6M HFD	
LVIDD, cm	3.187±0.07	3.237±0.078	3.078±0.033	3.229±0.048	0.93
LVIDS, cm	1.634±0.033	1.787±0.056	1.65±0.028	1.779±0.032	0.89
EF (%)	0.813±0.005	0.773±0.01	0.792±0.005	0.775±0.004	0.86
Heart Rate (bpm)	674±17	636±23	652±9	659±9	0.33
Heart Weight (g)	NA	0.176±0.008	NA	0.170±0.006	0.62

Numbers showed in table are Mean±SEM. P value compared 6 month HFD parameters.

### *Sprr3* promotes procollagen type I mRNA and protein synthesis in vascular smooth muscle cells in an Akt-dependent manner

**Fig 5:**
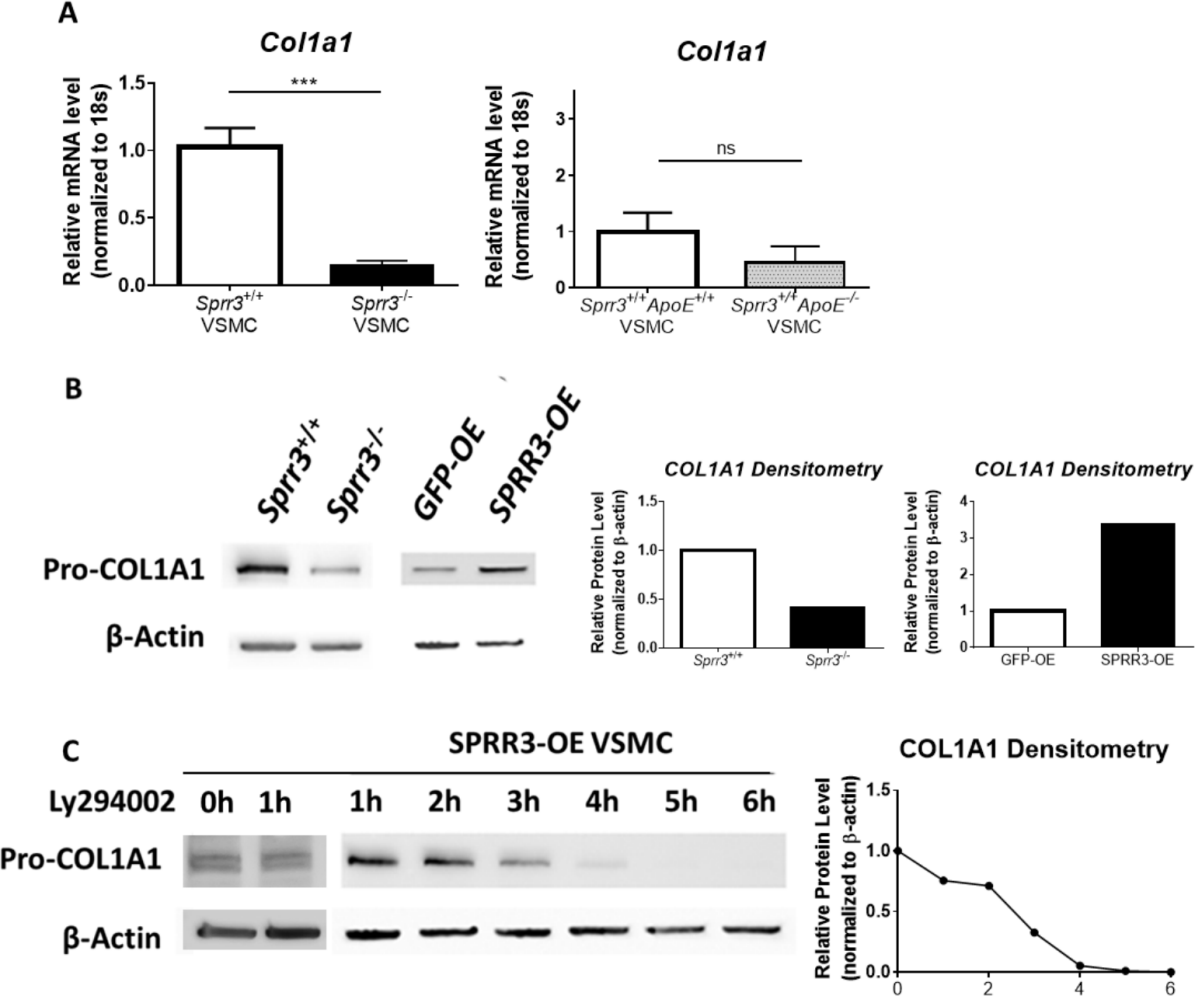
Collagen type I mRNA and protein expression are positively associated with SPRR3 expression in VSMCs *in vitro*. (A) Real time RT-PCR assessment of procollagen type 1α1 relative transcript levels in *Sprr3*-KO VSMCs compared to WT-VSMCs, and WT-VSMCs compared to *ApoE*-null VSMCs (B) Cell lysates from WT-VSMCs, *Sprr3*-KO VSMCs, VSMCs overexpressing vector control, and VSMCs overexpressing SPRR3 were subjected to immunoblot analysis using anti-procollagen type 1α1 or loading control anti-β-actin (~42kDa). Densitometry was performed to normalize protein levels to the loading control (C) Cell lysates from *SPRR3*-OE VSMCs that were treated with 25μM Ly294002 were collected at 1 hour intervals between 1 and 6 hours and were subjected to immunoblot analysis using anti-procollagen type 1α1 (n = 4*** p<0.001).

To determine whether the reduced collagen content observed in *Sprr3*-deficient lesion caps may be due to an *Sprr3*-dependent change in collagen synthesis, we tested whether collagen mRNA and protein levels were affected in primary vascular smooth muscle cells (VSMCs) isolated from *Sprr3* knockout (*Sprr3*^-/-^ VSMCs) or wild-type mice (*Sprr3*^+/+^ VSMCs) (Fig 5A) Using real-time RT-PCR, we showed that, when compared with wildtype VSMCs, *Sprr3*^-/-^ VSMCs contained significantly less (~80% reduction) procollagen type I α I transcripts (Fig 5A). There was no statistically significant difference between wild-type VSMC and ApoE-null VSMC collagen expression (Fig 5A). These data were further confirmed by evaluating protein expression using cell lysates from primary *Sprr3*^-/-^ and wildtype VSMCs by immunoblotting for collagen type I protein levels (Fig 5B). Furthermore, we utilized VSMCs previously generated in our laboratory to overexpress human SPRR3 (SPRR3-OE) or empty vector (GFP-OE) [15, 20], and demonstrated that overexpression of SPRR3 in VSMCs resulted in an increase in intracellular procollagen protein expression as compared to vector transduced control VSMCs (Fig 5B). We recently observed a PI3K-dependent increase in active Akt in association with *SPRR3* expression in VSMCs and showed that the survival advantage from *SPRR3* expression in VSMCs is abrogated in the presence of a PI3K inhibitor [20]. To determine if procollagen expression was dependent on PI3K/Akt signaling we treated *SPRR3* overexpressing VSMCs with PI3K inhibitor Ly294002 and observed reduced procollagen type I α I protein levels in *SPRR3* overexpressing VSMCs as detected by immunoblot (Fig 5C).

### MMP2 transcript and activity levels are increased in *Sprr3* deficient VSMCs in an PI3K/Akt-independent manner

**Fig 6:**
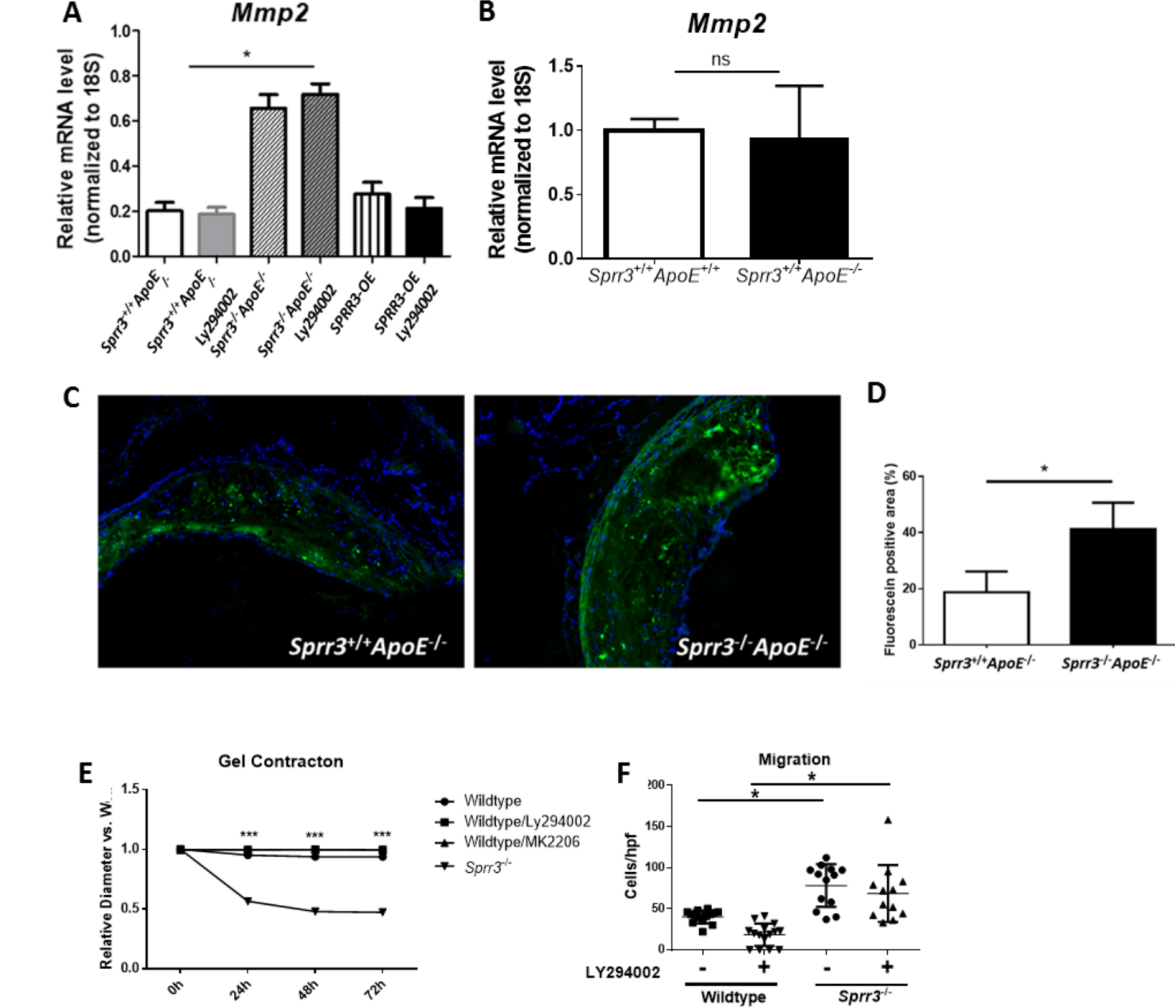
VSMC expression of MMP2 transcripts and matrix remodeling activity are increased with *Sprr3* loss. (A) Relative transcript levels of MMP2 were assessed in *Sprr3*^*+/+*^*ApoE*^*-/-*^ VSMCs, *Sprr3*^-/-^*ApoE*^-/-^ VSMCs, and VSMCs overexpressing *SPRR3* that were untreated or treated with 25μM Ly294002 (PI3K inhibitor, PI3KI) by real time RT-PCR. N = 4 independent experiments. (B) Relative transcript levels for WT and *Sprr3*^+/+^*ApoE*^-/-^ VSMCs were also assessed. (C) Representative images of aortic root *in situ* gelatinase zymography from *Sprr3*^+/+^*ApoE*^-/-^ or *Sprr3*^-/-^*ApoE*^-/-^ (DKO) mice. Green = fluorescein (cleaved gelatin). Blue = DAPI. (D) Graph of quantification of fluorescent signal localized to plaque area of *Sprr3*^+/+^*ApoE*^-/-^(n = 8) and *Sprr3*^-/-^*ApoE*^-/-^ (n = 8) and. * p < 0.05. (E) Gel contraction assays were performed in wildtype VSMCs with two PI3K inhibitors, Ly294002 and MK2206, and in *Sprr3*^-/-^ VSMCs. VSMCs were polymerized in collagen matrices, which were released after gelling and areas measured at designated time points. Matrix contraction (starting-final) shown as averages±SD for triplicate samples. Data represent mean±SD of 4 separate experiments, Gel contraction data analyzed using two-way ANOVA, *p < p<0.001. (F) Transwell migration assays were performed with wildtype and *Sprr3*^-/-^ VSMCs with and without PI3K inhibitor, Ly294002. The number of cells migrated to the bottom of an 9 μm pore membrane in response to media containing 10% FBS were assessed after 5 hours by counting migrated cells stained with crystal violet. Data represent mean±SD of 4 separate experiments, data were analyzed using the one-way ANOVA/Kruskal-Wallis. *p<0.001.

Because two opposing metabolic processes determine the net amount of collagen composing a plaque, we evaluated whether *Sprr3* loss reduced cap size by also by increasing collagen degradation through MMP activity. MMPs generally colocalize with macrophages/foam cells and also with VSMCs [29]. Moreover, MMPs are likely involved in plaque rupture [9]. To determine whether expression of the major VSMC-associated MMPs (MMP2, 3 and 9) were upregulated in *Sprr3*-deficient cells and if *Sprr3*-dependent changes in MMP expression were PI3K/Akt dependent, mRNA was collected from primary *Sprr3^+/+^ApoE^-/-^* VSMCs, *Sprr3*^-/-^*ApoE*^-/-^VSMCs, and *SPRR3*-OE VSMCs either untreated or treated with 25μM of the PI3K inhibitor (Ly294002). Transcripts for *Mmp2* were increased in *Sprr3* deficient VSMCs ~2.3 fold over WT-VSMCs, and this increase was abrogated in VSMCs stably transduced to overexpress *SPRR3* to levels similar to control transduced cells (Fig 6A, n = 4 independent experiments). *Mmp2* levels were unaffected by *ApoE* ablation (Fig 6B). Transcripts for MMPs typically associated with macrophages, such as MMP8 and 13, were undetectable by real time RT-PCR in both WT and KO VSMCs. Unlike the down regulation of procollagen mRNA and protein levels in *Sprr3* deficient cells, the enhanced expression of *Mmp2* transcripts in *Sprr3*-KO VSMCs was not sensitive to PI3K inhibition (Fig 6A).

MMP2 is a 72 kDa member of the gelatinase subfamily of MMPs [30]. Therefore, we tested if *Sprr3* loss in *ApoE*-deficient mice resulted in an increase in lesion-associated gelatinase activity. When aortic root cryosections were subjected to *in situ* zymography with a gelatin substrate, foci of fluorescence were evident within lesions (Fig 6C, n = 8). The data obtained from multiple sections of aortic roots of each experimental animal were quantified and averaged to demonstrate that *Sprr3* loss resulted in a statistically significant increase of ~2-fold in intralesional gelatinase activity (Fig 6D, n = 3 sections from each root; n = 8 animals, data represent mean±SD).

Matrix remodeling/reorganization was further tested using an *in vitro* model of matrix contraction [31, 32]. To further strengthen the possibility that *Sprr3* enhanced expression and activation of proteolytic proteins and thereby collagen reorganization, we embedded WT or *Sprr3*-deficient VSMCs in 3D type I collagen gels and observed gel contraction over 72 hours. *Sprr3* loss significantly enhanced collagen gel contraction. The addition of two distinct PI3K/AKT pathway inhibitors did not alter the phenotype observed with *Sprr3* loss (Fig 6E, n = 4, ***p<0.001 compared to WT).

### Loss of *Sprr3* increases VSMC migration independent of PI3K/Akt

Enhanced VSMC migration has been linked to neointimal progression and atherosclerosis progression [33]. We evaluated whether VSMC migration was also regulated by *Sprr3*, and whether this activity was dependent on PI3K/Akt signaling. We utilized the Boyden Chamber assay to evaluate whether *Sprr3* had an effect on migration (Fig 6F). We found that the absence of *Sprr3* resulted in a significant ~2-fold increase in migration in the presence or absence of the PI3K inhibitor Ly294002, and therefore, was independent of this signaling pathway (n = 3 independent experiments).

### Loss of *Sprr3* in VSMCs leads to reduced p38 and increased pERK signaling

**Fig 7:**
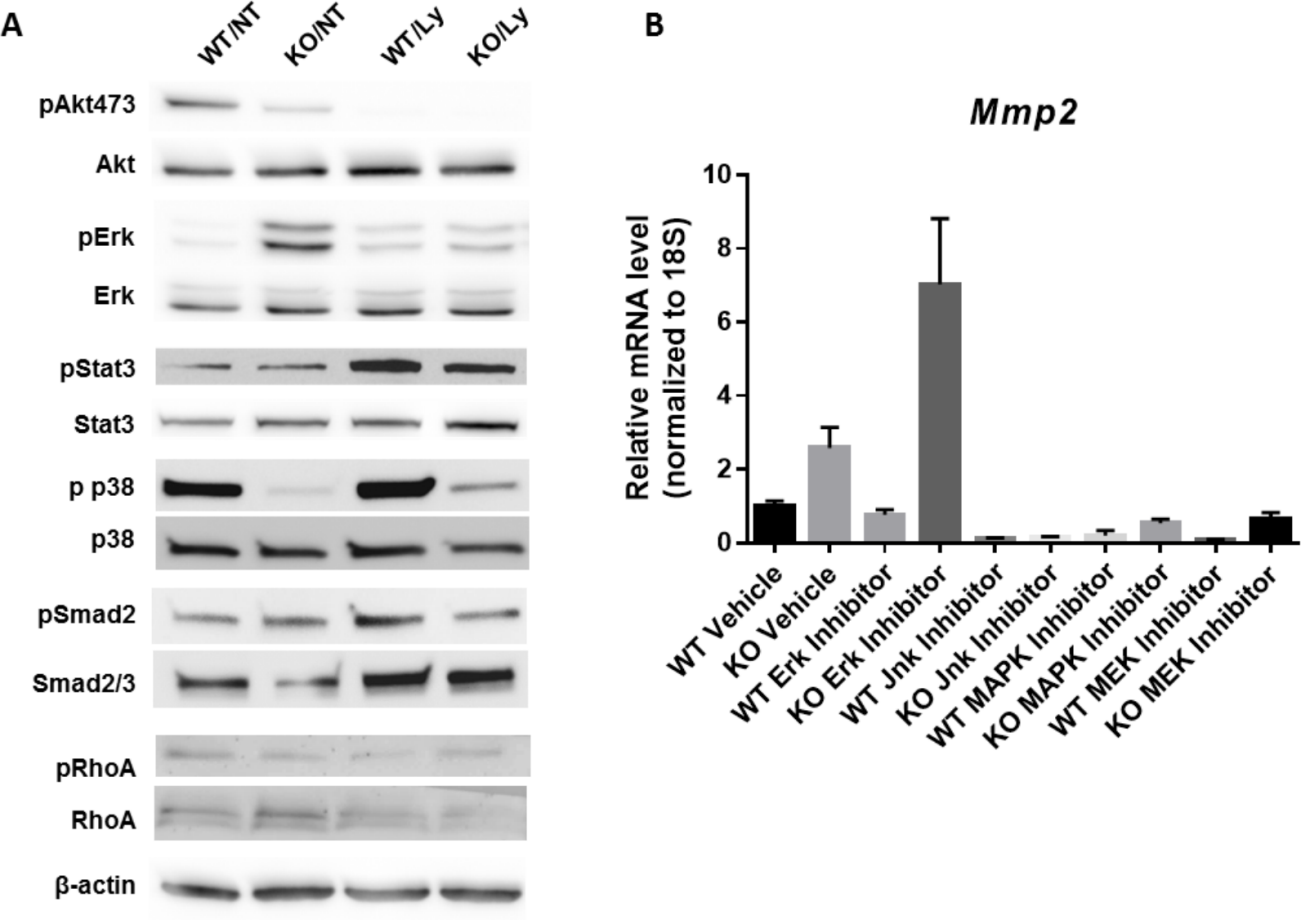
*Sprr3*^-/-^ VSMCs display altered cell signaling. Wildtype (WT) and *Sprr3*^-/-^ (KO) VSMCs were not treated (NT) or treated with PI3K inhibitor, Ly294002 (Ly). Representative immunoblots from 3 independent experiments are shown for phosphorylated and total Akt, Erk, Stat3, p38, Smad2 and RhoA. Beta-actin served as a loading control. (B) WT and KO VSMCs were treated with inhibitors to Erk, Jnk, MEK and p38 MAPK and assessed by RT-PCR for *Mmp2* expression levels. These were normalized to 18S.

We have identified several important *Sprr3*-modulated VSMC cellular phenotypes, some of which were dependent and some independent of PI3K/Akt signaling. To examine additional signaling pathways that may explain the Akt/PI3K independent regulation of cellular behavior, we examined changes in activation of several pathways by immunoblot primary VSMCs isolated from WT vs. *Sprr3*-deficient mice (representative figures from n≥3 experiments). We observed opposing regulation of the mitogen-activated protein (MAP) kinase, ERK and p38, with *Sprr3* loss (Fig 7A), pathways that have been associated with VSMC migration [34]. *Sprr3*-KO VSMCs demonstrated significantly increased pERK but significantly decreased p-p38 activation as compared to WT-VSMCs and these effects were present even with PI3K/Akt inhibition. Both STAT3 and Rho kinase have also been implicated in VSMC migration and contraction [35]. However, pSTAT3 and pRhoA were not significantly different between WT vs. *Sprr3*-deficient VSMCs. In addition, there was no change in pSmad2 levels, which is an important downstream molecule in the TGFβ signaling pathway [36]. Treatment of WT and KO VSMCs with inhibitors of Erk or MAPK pathway inhibitors did not affect *Mmp2* expression patterns (Fig 7B); suggesting that other yet unknown pathways may be involved in the SPRR3-mediated upregulation of gelatinase activity.

## Discussion

Plaque instability and rupture with thrombotic occlusion underlie most cases of acute myocardial infarction and sudden coronary death [37]. Fatal complications of atherosclerosis do not occur as a result of vessel stenosis due to excessive lesion burden but rather due to thrombotic complication at the site of plaque rupture or erosion [1, 14]. Here we report that loss of *Sprr3* in *ApoE*^-/-^ mice leads to increased atheroma vulnerability and all of the major features of plaque destabilization and rupture. After 6 months on a high-fat diet, *Sprr3*^-/-^*ApoE*^-/-^ deficient mice exhibited atherosclerotic plaques with increased necrotic core size, reduced cap collagen content, and reduced VSMC content in both brachiocephalic artery and aortic root lesions. Furthermore, they developed evidence of intraluminal thrombi and coronary artery lesions with advanced features, including intraplaque hemorrhage and spontaneous left ventricle microinfarcts.

Previously, we identified SPRR3 expression as evident exclusively in plaque-associated VSMCs in both human and mouse atherosclerotic lesions. Using bone marrow transplant experiments coupled with direct measurements of inflammatory cells and cytokines, we demonstrated that the effects of *Sprr3* on disease progression was not mediated by its effects on inflammatory cells or altered chemokines but rather mediated by vascular cells [15, 20]. Moreover, by demonstrating that *Sprr3* mRNA and protein expression were not detected in endothelial cells and that neither proliferation rate nor survival was affected in *Sprr3*-deficient endothelial cells, we attributed *Sprr3*’s effects on atherosclerosis to VSMCs [38]. Taken together, *Sprr3*’s effects on atheroma progression are believed to be mechanistically driven by VSMCs [15, 20].

Several earlier reports have described mouse models exhibiting key features of plaque instability (i.e. increased apoptosis of VSMCs, decreased cap collagen content, buried fibrous caps and increased plaque inflammation), in *ApoE*^-/-^ mice on high fat diets (HFD) [39–42]. Importantly, these models lack direct evidence of rupture and are primarily driven by mechanisms that involve inflammation and/or hyperlipidemia [39–42]. Our studies demonstrated that *Sprr3*^-/-^*ApoE*^-/-^ (DKO) mice on 6 months of HFD exhibited lesions in aortic root, brachiocephalic and coronary arteries containing features that model our current understanding of unstable plaques including larger, lipid-rich necrotic (thrombogenic) cores with thinner fibrous caps that have reduced levels of both VSMCs and collagen content [14, 43]. Indirect evidence of rupture, such as intraplaque hemorrhage [44], were observed in some advanced coronary artery lesions only in DKO animals. Direct evidence of plaque rupture, in part due to its stochastic nature, has rarely been reported in mice [1, 14]. Thus, it is highly significant that lesion disruption and early thrombus formation were observed in six out of 24 mouse brachiocephalic arteries in the DKO mice after 6 months HFD. We also identified evidence of a fibrin- and platelet- rich organized thrombus within the brachiocephalic artery in one of 24 animals studied. Although DKO mice contained significantly higher numbers of advanced coronary artery lesions containing large necrotic cores and other features of instability, we did not come across any sections with direct evidence of plaque rupture and early thrombus formation in coronary arteries. The majority of the DKO mice also exhibited ischemic microinfarcts in the heart, suggestive of embolization of a very small fragment of ruptured plaques; the presence of small ischemic infarcts in the left ventricle did not diminish overall cardiac function by echocardiography. The absence of large mural infarcts (any occurrence of spontaneous death during the 6 month of study) taken together with the lack of direct evidence of coronary lesion rupture may suggest that significant and major coronary lesion rupture may be rare in this model. One speculation as to why we observed ruptured lesions in the brachiocephalic but not coronary arteries may be due to differences in hemodynamics of coronary blood flow in mice vs. humans.

Mechanisms of lesion vulnerability have focused attention on thinning of the fibrous cap, which overlies the lipid core. Two main hypotheses address matrix homeostasis of the fibrous caps including matrix synthesis, which is controlled by VSMC numbers and production capacity, as well as matrix proteolysis and remodeling [44]. Interestingly, *Sprr3* loss contributed to diminished fibrous cap (and resulting plaque instability) via all of these mechanisms. We demonstrated in an earlier study that *Sprr3* loss resulted in reduced VSMC numbers in advanced lesions by via increased susceptibility to apoptosis [20]. In this study, we demonstrated that *Sprr3* loss also resulted in reduced synthesis of both mRNA and protein of procollagen type 1, the main matrix constituent of the fibrous cap [44], in a PI3K/Akt dependent manner. Regulators of VSMC collagen synthesis have not been extensively explored, although most studies point to TGF-β as a critical player [45]; however, we did not observe any changes in pSMAD2 signaling with *Sprr3* loss. A few studies have suggested mechanisms by which PI3K/Akt may regulate collagen synthesis [46–48]. In hepatic stellate cells, PDGF treatment increases focal adhesion kinase (FAK) activity, which leads to increased PI3K/Akt activity, and promotes collagen type I mRNA and protein production [49].

Additionally, *Sprr3* loss resulted in higher MMP2 expression in VSMCs, which resulted in increased matrix remodeling as demonstrated in aortic root lesions. Matrix remodeling is implicated in plaque destabilization and rupture [50]. Although it is believed that inflammatory cells within the plaques are the major source of MMPs and other proteases, VSMCs also produce matrix degradation enzymes [9, 29, 50]. In fact, SPRR3 loss did not alter MMP3 or 9 (which are typically macrophage derived) transcript levels in VSMCs. The MMP up-regulation seen in *Sprr3* deficient VSMCs in culture suggested a direct regulation by *Sprr3* that was not secondary to changes in nitric oxide, vascular biomechanics or ROS. Post mortem examination of human coronary arteries revealed increased immunostaining of MMP 2 and 9, and elevated MMP2 activity in the plaques of highly remodeled segments [9, 29, 50, 51]. Gelatinolytic activity has also been found localized to atherosclerotic lesions and MMP2 has been implicated in atheroma progression in genetic models [52]. On the other hand, reports have also found MMP2 to be protective [53]. *Sprr3*-deficient VSMCs exhibited enhanced capacity for matrix remodeling in an *in vitro* gel contraction assay as well as increased migration. Interestingly, MMP2 has been implicated in enhanced VSMC migration [54]. Importantly, *Sprr3* modulation of MMP expression and activity, collagen gel remodeling *in vitro*, and VSMC migration were all PI3K/Akt independent. Enhanced migration has been linked to intimal thickening and increased lesion size; however, we are not aware of any association with plaque stability [55].

Previous studies have demonstrated that targeting solely VSMC apoptosis in the setting of atherosclerosis induced by HFD in *ApoE*^-/-^ mice can generate key features of unstable plaques, including thin fibrous caps [56, 57]. Other elegant reports not only further support the role of VSMC survival in atherogenesis but directly implicate *Akt1*’s role [11, 13]. Whereas *Akt2* and *Akt3* primarily modulate atherogenesis by controlling macrophage biology [58], a recent study has shown that targeted deletion of *Akt1* in VSMCs results in larger necrotic core, enhanced VSMC apoptosis within lesions, reduced fibrous cap and collagen content [13]. Notably, although global deletion of *Akt1* resulted in a much more dramatic disease phenotype characterized by cardiac dysfunction resulting from large ischemic LV infarcts (presumably from ruptured plaques), targeted deletion of *Akt1* in VSMCs resulted in a relatively modest phenotype, suggesting that *Akt1* loss in endothelial cells serve as the primary driver of the increased atherosclerosis observed in mice lacking *Akt1* [11, 13]. These studies evaluating *Akt1* in VSMCs did not report whether collagen synthesis was affected but linked the reduced cap thickness solely to reduced VSMC numbers [13]. Based on our data, we would anticipate that *Akt1* may be regulating plaque collagen content in part by downregulating VSMC collagen synthesis.

A conspicuous feature of the *Sprr3*-deficient model is the lack of increase in inflammation. Whereas abrogating VSMC survival (by inducing experimental apoptosis) within lesions was accompanied by increased cellular inflammation and serum IL-6, abrogating *Akt1* in VSMCs, which also increased intra-lesional VSMC apoptosis, did not increase cellular infiltrate or expression of inflammatory cytokines [13, 56, 57]. Moreover, loss of *Akt1* did not alter expression of MMP2 or 9 [13]. This is consistent with our findings, which suggest that the upregulation of MMP2 and the matrix remodeling effects of *Sprr3* were PI3K/Akt independent.

Taken together, our data indicate that the effects of *Sprr3* on plaque progression and instability are likely due to reduced cap strength. SPRR3 loss reduces cap VSMCs [20], decreases collagen content (via reduced VSMCs and reduced matrix synthesis), and shifts fibrous cap homeostasis from matrix accumulation to matrix loss by simultaneously promoting matrix degradation/remodeling via increasing MMP2. Given the modest phenotype of VSMC-specific loss of *Akt1* [13], VSMC apoptosis and diminished collagen synthesis in the *Sprr3*-deficient mice, both of which are PI3K/Akt-dependent, are unlikely to be the sole drivers of the *Sprr3* phenotype. Hence, other signaling pathways that are also affected by *Sprr3* loss are likely necessary for the plaque instability. We have identified that *Sprr3* loss increased pERK activity but decreased p38 activity in VSMCs. Both pathways have been linked to VSMC migration and response to angiotensin II and TNFα [59, 60]. However, neither pathway has been directly linked mechanistically to VSMC-mediated plaque instability, although p38 has been shown to regulate vascular inflammation [1, 31, 43, 44]. Understanding the precise molecular mechanism of SPRR3 action may help us to better target specific VSMC cell behaviors that regulate fibrous cap homeostasis. These findings lead us to propose that *Sprr3* represents a modulator of atheroma-cap structural integrity that affects VSMC number, collagen production and matrix degradation, and that the *Sprr3*^-/-^*ApoE*^-/-^ mouse represents the first model that affects all of these structural components simultaneously.

## Methods

### Animal studies and histology

All studies were approved by the Vanderbilt University Institutional Animal Care and Use Committee. All methods were performed in accordance with the relevant guidelines and regulations. Mice were maintained at Vanderbilt University Vivarium and were previously described [26]. Briefly, a targeting vector was designed containing a Neo cassette flanked by LoxP and FRT sites carrying homology arms to the Sprr3 allele. Recombineering was used to introduce the targeting vector into the 129/C57 ES cell line, cells were selected with neomycin and injected into blastocysts for implantation. This was performed by InGenious Targeting Laboratories. Chimeras were bred to homozygosity, the neomycin cassette was removed by cross with the ACTB-Flp mice (Jackson Laboratories), then mice were backcrossed 15 generations onto the C57Bl/6 background (Jackson Laboratories). *ApoE*^-/-^ mice were obtained from Jackson Laboratories on a C57Bl/6 background. Mice were sacrificed by overdose with isofluorane (HenrySchein).

Female *Sprr3*^+/+^*ApoE*^-/-^ and *Sprr3*^-/-^*ApoE*^-/-^ (DKO) mice on C57Bl/6 background were utilized for this study. Mice were maintained on high fat diet for six months. Echocardiograms were performed at 2, 4 and 6 months and were blindly read using short axis and parasternal long-axis views with the leading-edge method. Brachiocephalic artery sections (10 µm) were collected from *Sprr3*^+/+^*ApoE*^-/-^ and *Sprr3*^-/-^*ApoE*^-/-^ mice fed high fat diet for six months and stained with Masson’s trichrome. Morphometry calculations were performed using ImageJ (NIH). The relative lipid-rich core area was determined by dividing the area of the lipid-rich core, as defined by the colorless area within the lesion, by the total area of the lesion. Percent cap collagen content was determined by dividing the area of connective tissue along the luminal surface of the lesion by the total cap area, which was determined by measuring the area between the luminal surface and the lipid-rich core [61]. For evaluation of lipid-rich core size, cap thickness, and cap collagen content, five sections per mouse were assessed. Sections not containing lesions with detectable lipid-rich core or fibrous cap were discarded, and the remaining images were quantified as described. For quantification of cap macrophage content, brachiocephalic artery atheroma sections from 6-month-old, high fat diet-fed *Sprr3*^+/+^*ApoE*^-/-^ and DKO mice were immunohistochemically stained with antibody against F480. Using ImageJ, the area of F480+ staining was measured and normalized against cap area to determine % macrophage area within the cap.

Hearts were collected from SPRR3^+/+^*ApoE*^-/-^ and DKO mice and were divided into three large transverse sections before being paraffin embedded for serial sectioning. A single 10μm section from each large transverse section was then MOVAT stained and left coronary arteries were categorized according to Stary’s classification [62, 63] as no lesion, fatty streaks (AHA type II), intermediate atheromas (AHA type IV), or advanced atheromas (AHA type V). In brief, lesions categorized as fatty streaks were characterized by foam cell accumulation as represented by a small amount of blue mucin-type buildup on the luminal surface of the artery. Lesions categorized as intermediate atheromas were characterized by pooled lipid/foam cells, represented by colorless areas associated with the blue mucin buildup within the lesion and are covered by a thin intimal layer of cells. Lesions categorized as advanced atheromas are more complex, with well-delineated lipid-rich cores and thicker caps. Each heart, in addition to apical sections, was also evaluated at four additional levels spanning from the apex to base. These sections were stained with Masson’s trichrome for evaluation for areas of infarct. Measurements of the length of the entire endocardial circumference and that segment of the endocardial circumference made up by the infarcted portion from each of the four slices of the left ventricle were determined. The infarct size, expressed as a percentage of the left ventricle, was calculated by dividing the circumference of the infarct by the total circumference of the left ventricle.

For analysis of the aortic root lesions, the aortic root was collected then embedded in OCT media. 10 μm thick cryosections were cut, then fixed and stained with Masson’s trichrome. Images were captured then analyzed using Image J software (NIH). Fibrous caps were defined as the VSMC and proteoglycan rich area that overlayed the necrotic cores, defined as cholesterol-rich, matrix-poor, acellular regions [11].

Detection of plasma IL-1β and IL-6 were performed by ELISA using ELISA kits (Bender). The procedure was done according to package instructions. The absorbance was read at 450 nm by microreader (Bio-Rad, 3550-UV) within 30 min of stopping the reaction with a wavelength correction at 570 nm.

Bleeding time was measured after anaesthetizing and placement on warming pad. A transverse incision was made with a scalpel over a lateral tail vein at a position where the diameter of the tail is ~2.3 mm. The depth of the incision was designed to lacerate the tail vein. The tail vein was hung over the edge of the table and immersed in normal saline in a hand-held test tube. The time from the incision to cessation of bleeding was recorded as the bleeding time. Platelet count was measured by Sysmex K-1000 automated hematology counter by the Vanderbilt Tissue Research Core Laboratory. Tissue factor expression was measured by flow cytometry (FACS analysis on a LSMII flow cytometer and FACS Diva v5.2 software, Becton Dickinson) using anti tissue factor antibody (rabbit affinity purified polyclonal from R&D systems/goat anti rabbit IgG conjugated to Cy3). The negative control was generated by incubating cells with rabbit IgG followed by goat anti secondary. Nonviable cells identified by 7AAD (molecular probes) staining and excluded from analysis.

### Vascular smooth muscle cell isolation

Smooth muscle cells were isolated as previously described [26]. Briefly, aortas were isolated between the aortic arch and iliac bifurcation. Fat, connective tissue and the endothelium were removed. Tissue was digested with collagenase and elastase. Cells were maintained in DMEM supplemented with 15% fetal bovine serum and antibiotics (pen/strep) under standard culture conditions.

### Transwell migration assay

Human fibronectin was diluted in phosphate buffered saline. Using a 24-well dish with transwell inserts, the fibronectin solution was used to coat both sides of the filter. Filters were blocked with 1% bovine serum albumin in DMEM media for 1 hour at 37°C. VSMCs were serum-starved overnight in 1% fetal bovine serum (FBS) in DMEM. 10^4^ cells were seeded in the transwells and incubated for 5 hours at 37°C. Cells were fixed with formalin, then stained with 0.5% crystal violet/0.2M boric acid for 30 minutes at room temperature. Destaining was performed with water. Cells were counted per field of view.

### Contraction assay

For the floating gel matrix assay 0.9 mL collagen coating (1.67mg/mL collagen, 20mM HEPES, 44mM NaHCO3, 1X DMEM) was mixed with 0.1mL cells (3.3 x 10^5^ cells/mL) for a final concentration of 1.5mg/mL collagen. The cell/collagen mix was plated in 3-wells of a 48-well plate (10^5^cells/well) and incubated for 15–20 minutes until gelled. A 30G needle was used to free the sides of the gel from the wells. DMEM supplemented with 10% FBS was added to the wells. Treatments were as follows: 1ng/mL TGFβ (R&D systems, #76666-MB), 10μM LY294002 (Sigma, #L9908), 100nM Wortmannin (Santa Cruz Bio, #sc-3505), 2.5μM MK2206 (Santa Cruz Bio, #sc-364537). Plates were imaged at 24, 48 and 72 hours.

### Immunoblotting

For protein collection, cultured cells were washed twice with PBS and lysed using RIPA buffer supplemented with protease inhibitors and phosphatase inhibitors (Roche). Whole cell lysates were rocked 30 minutes at 4°C then spun 15 minutes at 13,000xg and the supernatant collected. Protein concentrations were determined using a bicinchoninic acid (BCA) kit (Thermo Scientific). Proteins were denatured 10 minutes at 95°C in SDS sample buffer before being resolved by SDS-PAGE electrophoresis and transferred onto a nitrocellulose membrane (PerkinElmer). Blots were blocked in 5% nonfat milk and probed with antibodies against collagen type I (MDBioproducts, #203002) or β-actin (Sigma, #A5441).

For the analysis of various signaling pathways, VSMCs were cultured to confluence in DMEM supplemented with 10% serum and antibiotics (1% Pen/Strep). They were serum starved in DMEM with antibiotics overnight, then treated with 10% serum with or without Ly294002 (25μM) for 1 hour. They were then washed twice with PBS, then scraped to collect cell pellets. These were lysed as indicated above in RIPA buffer. After quantification and denaturation, 30μg of protein was used for electrophoresis as above. Blots were blocked in 5% milk with tris buffered saline (TBS) and 0.05% Tween-20, then probed with the following antibodies in 1% milk TBST: pErk (cell signaling #4370), Erk (cell signaling #4695), pAkt473 (cell signaling #9271), Akt (cell signaling #9272), pStat3 (cell signaling #9145), Stat3 (cell signaling #12640), pSmad2 (cell signaling #3108), Smad2/3 (cell signaling #5678) p-p38 (cell signaling #9211), p38 (cell signaling #9212), pRhoA (SantaCruz), RhoA (ProteinTech Group). Secondary antibodies conjugated with HRP and ECL reagents were used to visualize the bands by chemiluminescence.

### Real time reverse transcription polymerase chain reaction

For real time RT-PCR measurements of MMP-2, MMP-9 and MMP-3 levels, SPRR3-KO and WT-VSMCs were plated at a density of 5x10^4^ cells/well in a 12-well plate. Cells were serum starved in 1% FBS overnight and allowed to recover in normal serum for 18 hours. A subset of cells was also treated with 25 μM PI3K inhibitor Ly294002 (Sigma). In the case of inhibitor treatment, cells were seeded at 300,000 cells/well in a 6-well plate, serum starved for 4 hours in 0% FBS, then treated with the MAPK Inhibitor Tocriset (R&D Systems) SB202190 (p38 MAPK inhibitor), U0126 (inhibitor of MEK1/2), SP600125 (inhibitor of JNK) or Erk inhibitor (SCH772985, Selleck Chem) for 1 hour. Total RNA was isolated using Trizol (Invitrogen) following the manufacturer’s instructions. cDNA was generated using iScript cDNA Synthesis kit (Bio-Rad) from 1μg RNA. The cDNA was then used for quantitative real-time PCR (real time qRTPCR) as described [21]. Primers used for real time qRT-PCR were procollagen type 1α1 forward (5’-GCC AGA TGG GTC CCC GAG GT -3’), procollagen type Ia1 reverse (5’-GGG GGT CCA GCA GCA CCA AC-3’), MMP2 forward (5’-GTC CCG AGA CCG CTA TGT-3’), MMP2 reverse (5’-GTT GCC CAG GAA AGT GAA-3’), MMP3 forward (5’-CAT GGA GAC TTT GTC CCT TTT GAT-3’), and MMP3 reverse (5’-CGT CAA AGT GAG CAT CTC CAT TA-3’).

### *In situ* gelatin zymography

Slides were prepared coated with DQ-gelatin (25μg/ml) substrate (2% sucrose and 0.02% sodium azide in PBS) with the serine protease inhibitor, aprotinin. In the presence of this inhibitor, protease activity from MMPs and other non-serine-dependent proteases became distinguishable. Cryostat sections of 10 μm thickness from aortic roots of *Sprr3*^+/+^*ApoE*^-/-^ and *Sprr3*^-/-^*ApoE*^-/-^ mice (n = 8) were prepared directly onto DQ-gelatin substrate coated slides at 4°C onto gelatin-coated slides. The tissue was allowed to incubate on the slide with and without inhibitor for 24 hours at 37°C prior to coverslip and fluorescent visualization. The relative quantities of protease activity in the tissues appeared as fluorescent signal. The intensity and area of signal was quantified using Metamorph image analysis tool. Three sections from each animal were evaluated to obtain a mean activity for that animal.

### Statistical analysis

Data in which two groups were compared were analyzed with Mann—Whitney U test to determine statistical significance. In experiments with multiple groups, Kruskal-Wallis one way analysis of variance followed by Mann Whitney U test to compare two groups. Statistical differences between groups taken as p<0.05.

## Supporting information

S1 Fig Expression of *Sprr3*. (A) Expression levels of Sprr3 transcripts by RT-PCR in WT (*Sprr3*^+/+^*ApoE*^+/+^) and KO (*Sprr3*^-/-^*ApoE*^+/+^) VSMCs by RT-PCR. (B) Expression levels of Sprr3 transcripts by RT-PCR in *Sprr3*^+/+^*ApoE*^-/-^ and *Sprr3*^-/-^*ApoE*^-/-^ VSMCs by RT-PCR. (C) Expression levels of *Sprr3* transcripts in aortas from *Sprr3*^+/+^*ApoE*^-/-^ mice with or without lesions by real time RT-PCR. (D) Immunofluroscence of brachiocephalic and coronary arteries with and without lesions in *Sprr3*^+/+^*ApoE*^-/-^ mice. *Sprr3*^-/-^*ApoE*^-/-^ brachiocephalic arteries serve as a negative control. Arrows indicate lesions.
(TIF)

S2 Fig Effects of *Sprr3* loss on plaque instability parameters of the aortic root. (A) Representative image of aortic lesions stained with Masson’s trichrome. Quantifications of fibrous cap, necrotic area and collagen content correspond to lesions from DKO and *ApoE*^-/-^ mice. (B-C) Data are expressed as the percentage of the positively lesion area. Data are expressed as the mean+ standard error of the mean. N = 9 mice per group. P<0.05 compared with *ApoE*^-/-^ group.
(TIF)

S3 Fig Cap macrophage content is unaffected by *Sprr3* loss in advanced brachiocephalic atheromas. Brachiocephalic artery tissue sections collected from (A) *Sprr3*^+/+^*ApoE*^-/-^ (n = 10) or (B) *Sprr3*^-/-^*ApoE*^-/-^ (n = 10) mice fed high fat diet for 6 months were probed with an antibody against F480. (C) Quantification of %F480^+^ cap area identified no significant difference between groups. Dashed line indicates lesion cap. Original magnification, x40 (A-B).
(TIF)

S4 Fig *Sprr3* loss does not result in upregulation of tissue factor on vascular cells. Fluorescence histograms of flow cytometric analysis of mouse primary VSMCs and endothelial cells isolated from *ApoE* null or DKO mouse aortas. Cells were incubated with anti-tissue factor (purified rabbit polyclonal/goat anti rabbit Ig-Cy3). The negative control (red) represents cells incubated with rabbit IgG/goat anti rabbit Ig-Cy3
(TIF)
