# Supplementary figures and images for: Loss of SPRR3 in ApoE-/- mice leads to atheroma vulnerability through Akt dependent and independent effects in VSMCs
Source: PLoS One. 2017 Sep 8;12(9):e0184620. doi: 10.1371/journal.pone.0184620 (PMC5590986; doi:10.1371/journal.pone.0184620)

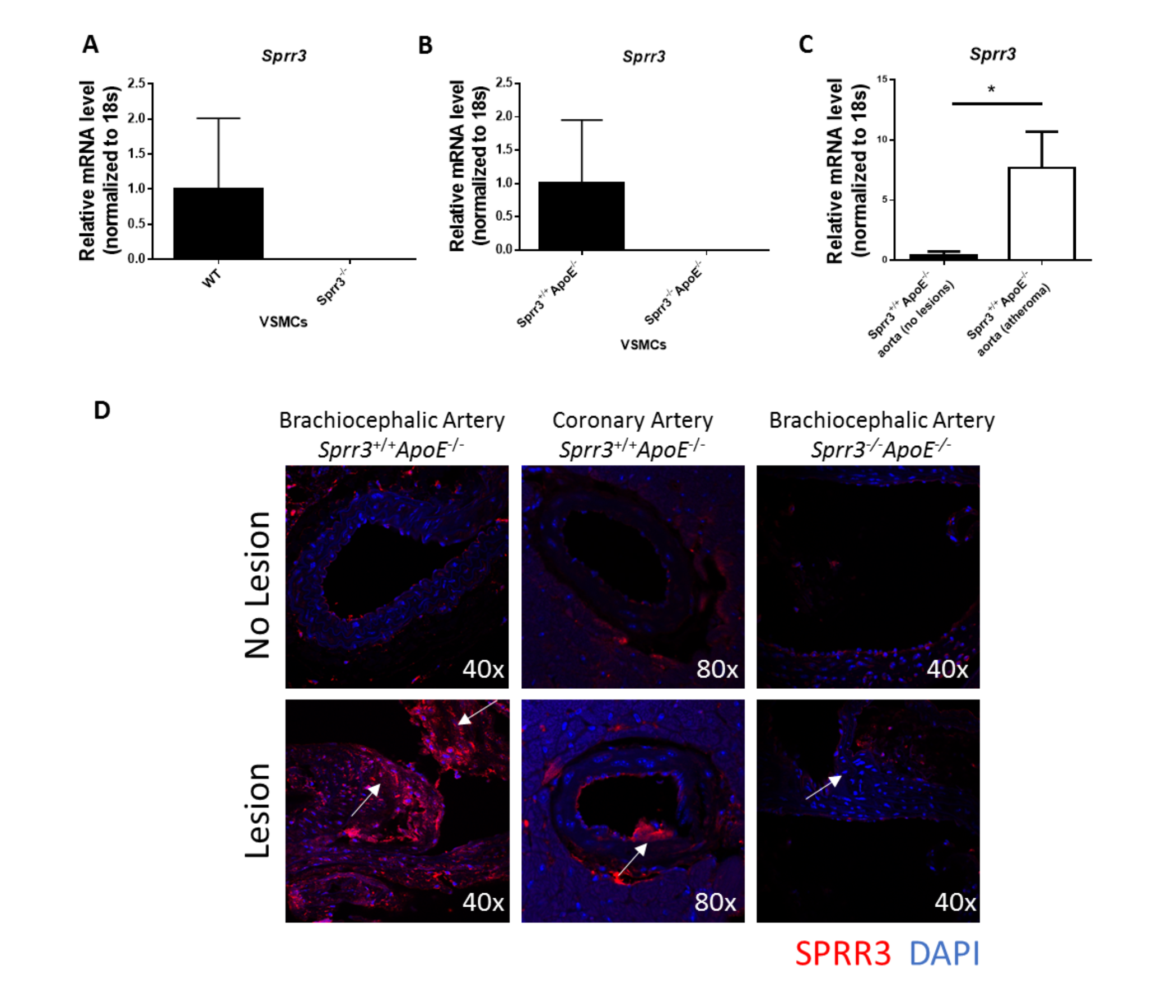

Supplement: S1 Fig — (A) Expression levels of Sprr3 transcripts by RT-PCR in WT (Sprr3+/+ApoE+/+) and KO (Sprr3-/-ApoE+/+) VSMCs by RT-PCR. (B) Expression levels of Sprr3 transcripts by RT-PCR in Sprr3+/+ApoE-/- and Sprr3-/-ApoE-/- VSMCs by RT-PCR. (C) Expression levels of Sprr3 transcripts in aortas from Sprr3+/+ApoE-/- mice with or without lesions by real time RT-PCR. (D) Immunofluroscence of brachiocephalic and coronary arteries with and without lesions in Sprr3+/+ApoE-/- mice. Sprr3-/-ApoE-/- brachiocephalic arteries serve as a negative control. Arrows indicate lesions. (TIF) [file pone.0184620.s001.tif]

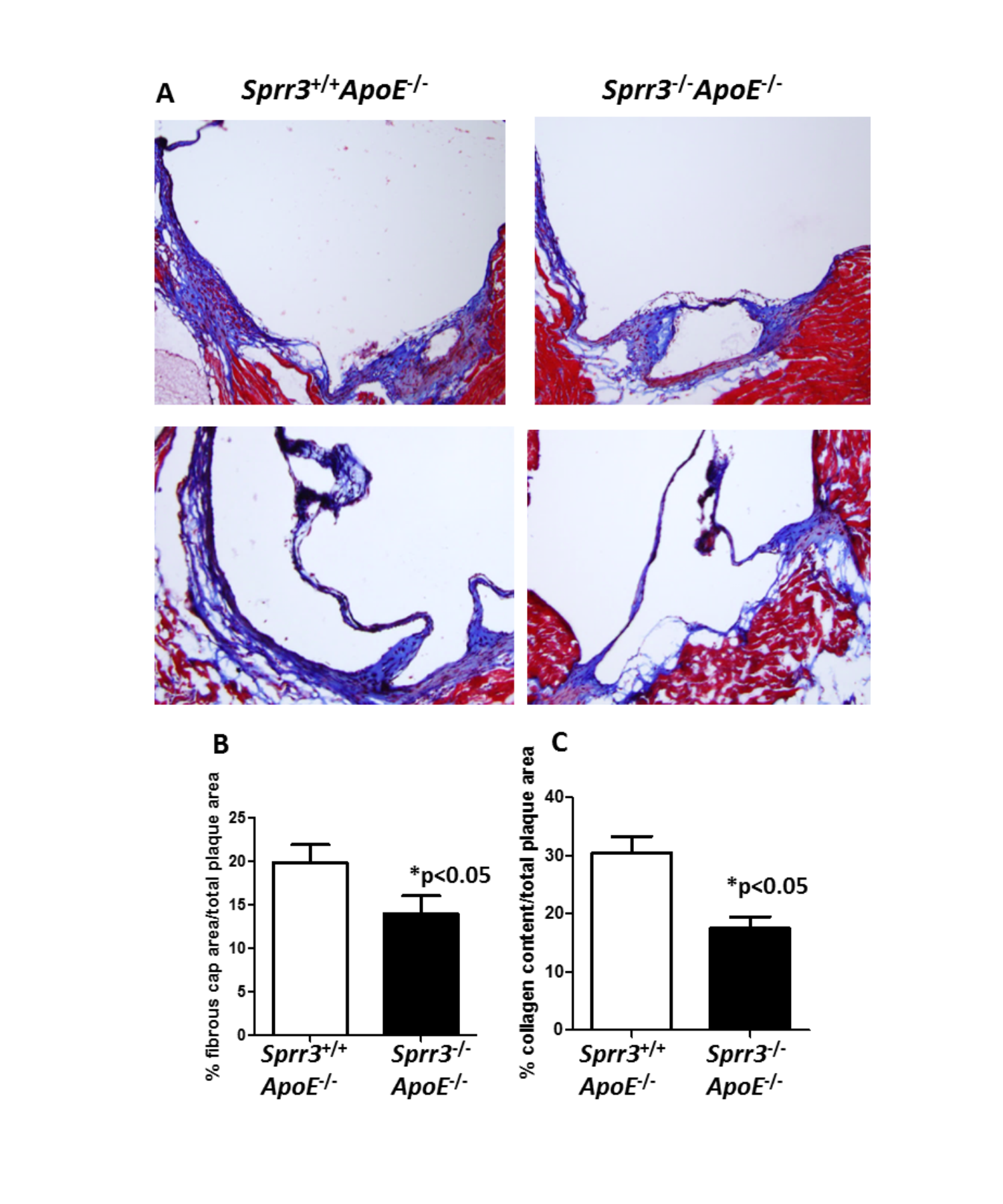

Supplement: S2 Fig — (A) Representative image of aortic lesions stained with Masson’s trichrome. Quantifications of fibrous cap, necrotic area and collagen content correspond to lesions from DKO and ApoE-/- mice. (B-C) Data are expressed as the percentage of the positively lesion area. Data are expressed as the mean+ standard error of the mean. N = 9 mice per group. P<0.05 compared with ApoE-/- group. (TIF) [file pone.0184620.s002.tif]

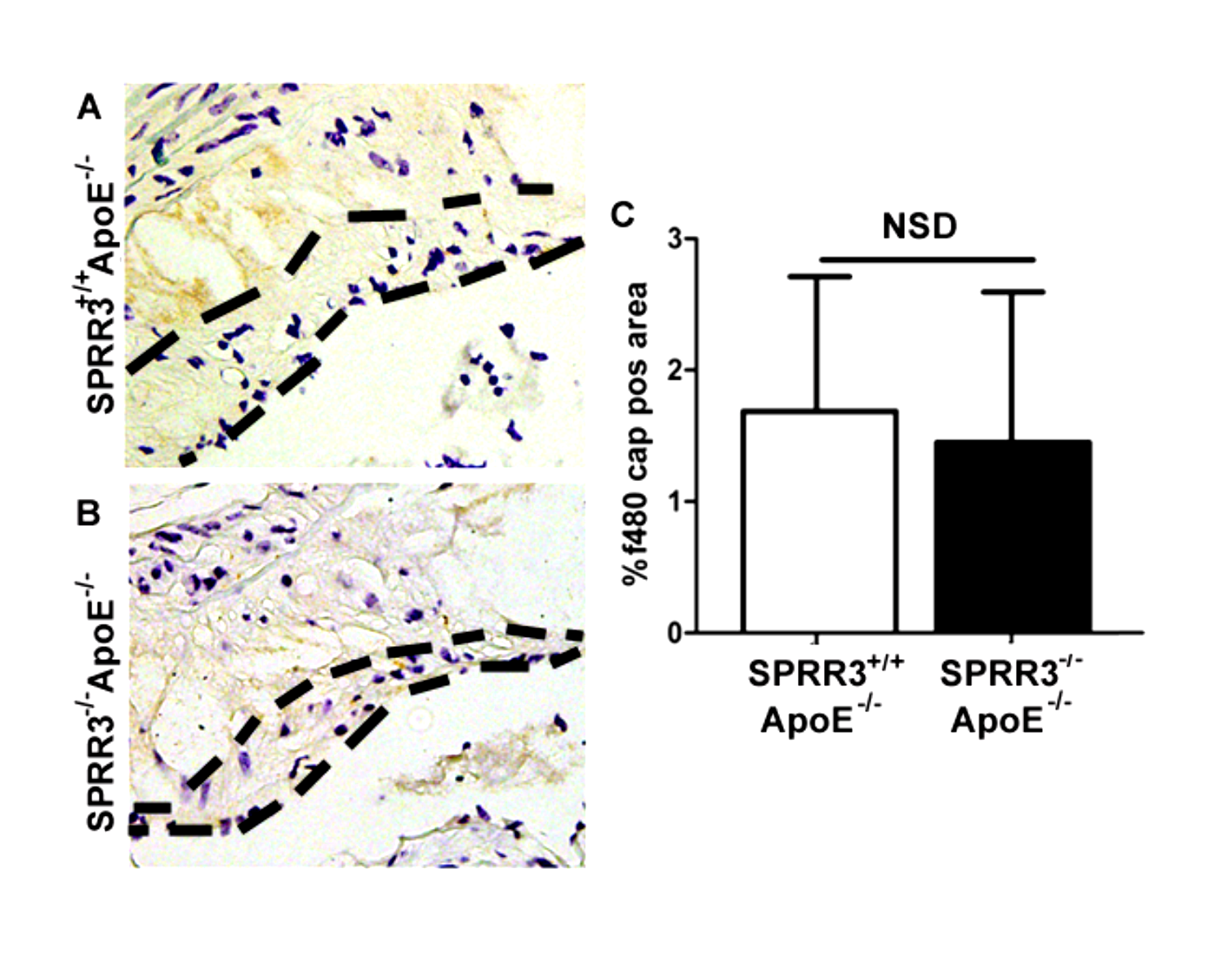

Supplement: S3 Fig — Brachiocephalic artery tissue sections collected from (A) Sprr3+/+ApoE-/- (n = 10) or (B) Sprr3-/-ApoE-/- (n = 10) mice fed high fat diet for 6 months were probed with an antibody against F480. (C) Quantification of %F480+ cap area identified no significant difference between groups. Dashed line indicates lesion cap. Original magnification, x40 (A-B). (TIF) [file pone.0184620.s003.tif]

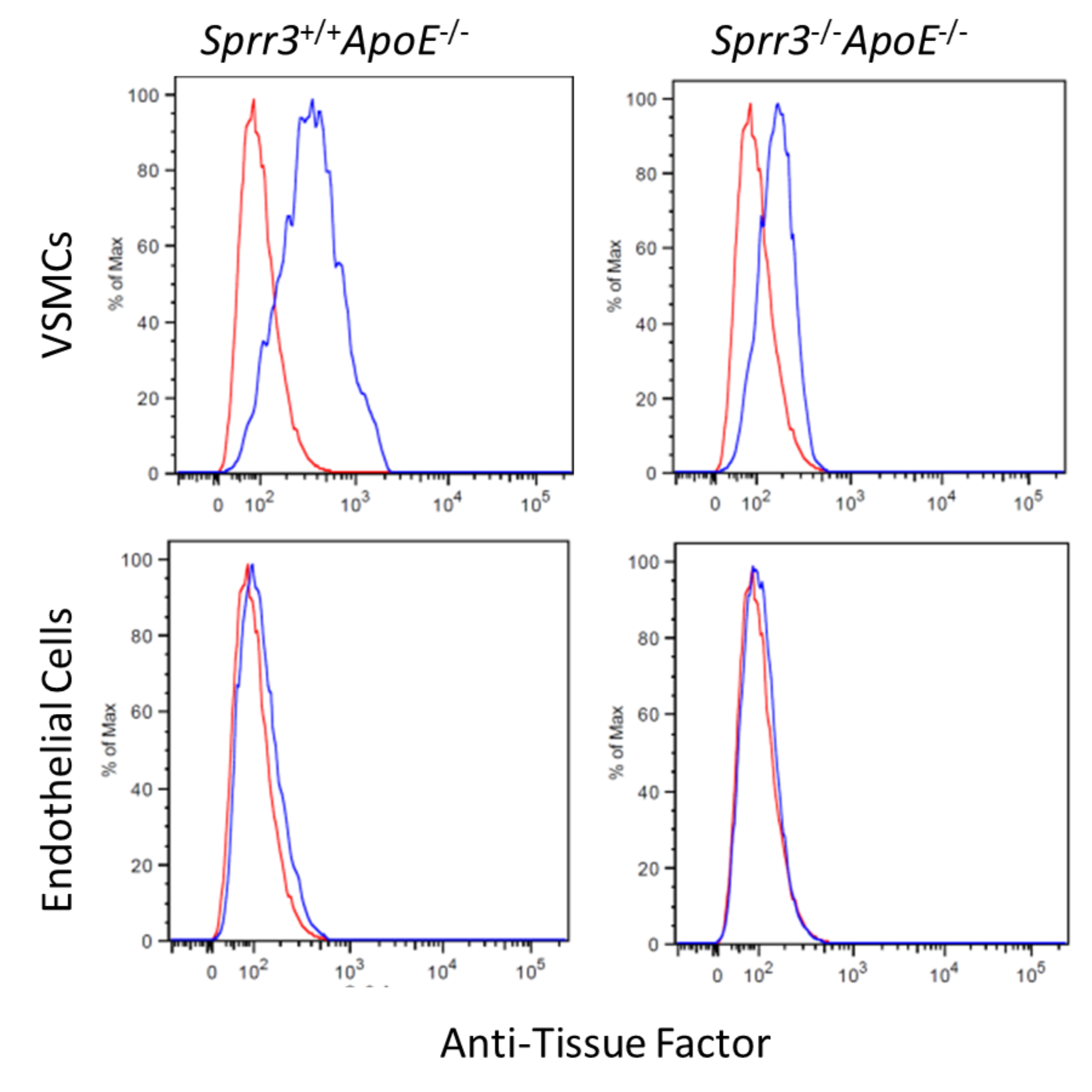

Supplement: S4 Fig — Fluorescence histograms of flow cytometric analysis of mouse primary VSMCs and endothelial cells isolated from ApoE null or DKO mouse aortas. Cells were incubated with anti-tissue factor (purified rabbit polyclonal/goat anti rabbit Ig-Cy3). The negative control (red) represents cells incubated with rabbit IgG/goat anti rabbit Ig-Cy3 (TIF) [file pone.0184620.s004.tif]
